# Reproducibility of a continuous ramp lower body negative pressure protocol for simulating hemorrhage

**DOI:** 10.14814/phy2.12640

**Published:** 2015-11-25

**Authors:** Victoria L Kay, Caroline A Rickards

**Affiliations:** Institute for Cardiovascular & Metabolic Diseases, University of North Texas Health Science CenterFort Worth, Texas

**Keywords:** Central hypovolemia, cerebral blood flow, ramp LBNP, repeatability, repeated design

## Abstract

Central hypovolemia elicited by application of lower body negative pressure (LBNP) has been used extensively to simulate hemorrhage in human subjects. Traditional LBNP protocols incorporate progressive steps in pressure held for specific time intervals. The aim of this study was to assess the reproducibility of applying continuous LBNP at a constant rate until presyncope to replicate actual bleeding. During two trials (≥4 weeks intervening), LBNP was applied at a rate of 3 mmHg/min in 18 healthy human subjects (12M; 6F) until the onset of presyncopal symptoms. Heart rate (HR), mean arterial pressure (MAP), stroke volume (SV), total peripheral resistance (TPR), mean middle and posterior cerebral artery velocities (MCAv, PCAv), and cerebral oxygen saturation (ScO_2_) were measured continuously. Time to presyncope (TTPS) and hemodynamic responses were compared between the two trials. TTPS (1649 ± 98 sec vs. 1690 ± 88 sec; *P* = 0.47 [*t*-test]; *r* = 0.77) and the subsequent magnitude of central hypovolemia (%Δ SV −54 ± 4% vs. −53 ± 4%; *P* = 0.55) were similar between trials. There were no statistically distinguishable differences at either baseline (*P* ≥ 0.17) or presyncope between trials for HR, MAP, TPR, mean MCAv, mean PCAv, or ScO_2_ (*P* ≥ 0.19). The rate of change from baseline to presyncope for all hemodynamic responses was also similar between trials (*P* ≥ 0.12). Continuous LBNP applied at a rate of 3 mmHg/min was reproducible in healthy human subjects, eliciting similar reductions in central blood volume and subsequent reflex hemodynamic responses.

## Introduction

Lower body negative pressure (LBNP) has been extensively utilized as an experimental technique to induce central hypovolemia and simulate hemorrhage in healthy, conscious humans (Wolthuis et al. 1974; Sander Jensen 1991; Convertino 2001; Cooke et al. 2004; Summers et al. 2009; Ward et al. 2010; Hinojosa-Laborde et al. 2014). It is well known that the progressively increasing LBNP results in decreased venous return, stroke volume (SV), cardiac output (CO), and mean arterial pressure (MAP), stimulating sympathetically mediated increases in heart rate (HR) and systemic vascular resistance (Wolthuis et al. 1974; Convertino et al. 2004; Cooke et al. 2004). LBNP also results in reductions of mean middle cerebral artery velocity (MCAv) and cerebral oxygenation (ScO_2_), which ultimately leads to presyncopal symptomology such as dizziness, nausea, and visual disturbances (Glaister and Miller 1990; Giller et al. 1992; Levine et al. 1994; Houtman et al. 2001; Guo et al. 2006). Traditionally, LBNP is applied in discrete, progressively decreasing steps, with each step lasting anywhere from 2 (Lewis et al. 2014) to 12 min (Convertino et al. 2006). However, application of LBNP with this stepwise approach may not accurately mimic actual volume loss (i.e., hemorrhage), as the cardiovascular system is able to compensate and stabilize when the negative pressure is held constant. In order to more accurately simulate *continuous* bleeding, we have implemented a ramp pressure profile with application of negative pressure at a continuous decompression rate of 3 mmHg/min.

Ramp LBNP profiles have been utilized in very few studies to date (Johnson et al. 1974; Balldin et al. 1996; Cooke et al. 2011). In these studies, continuous decompression elicited similar hemodynamic responses (i.e., reductions in MAP, SV, CO, MCAv, and increased HR) (Johnson et al. 1974; Balldin et al. 1996; Cooke et al. 2011) as those observed during stepwise LBNP profiles, but the reproducibility of these responses has not been reported. In contrast, a number of studies have assessed the reproducibility of not only tolerance to LBNP, but also the hemodynamic responses (Lightfoot et al. 1991; Convertino 2001; Howden et al. 2001; Lee et al. 2004). These investigators concluded that the tolerance and hemodynamic responses to stepwise LBNP was reproducible within subjects tested at varying time intervals from 3 days (Lightfoot et al. 1991) up to 1 year (Convertino 2001). As ramp pressure profiles may be utilized as a method to assess hemodynamic responses associated with continuous bleeding, it is important to determine the reproducibility of this experimental technique. Therefore, we tested the hypothesis that tolerance time and physiological responses to continuous LBNP applied at a rate of 3 mmHg/min would be reproducible in a cohort of young, healthy human subjects.

## Methods

### Subjects

Twenty-seven healthy, normotensive, nonsmoking subjects volunteered to participate in this study, conducted at the University of North Texas Health Science Center (UNTHSC) in Fort Worth, TX. The experimental protocol was reviewed and approved by the Institutional Review Board at UNTHSC. Prior to approval to participate in the study, each subject completed an orientation session, where a medical history was obtained and physical exam was performed, including seated and standing ECG and blood pressure measurements. Females underwent a urine pregnancy test and were excluded if pregnant; the pregnancy test was repeated immediately prior to experimentation. All female subjects were tested in the early follicular phase of their menstrual cycle (days 1–4), determined by self-report. Subjects were given a verbal briefing and written description of all the measurements and risks associated with the experiment, and were made familiar with the laboratory, personnel, procedures, and monitoring equipment. Each subject gave written informed consent to participate in this study. Because of the potential effects on vascular volume and cerebrovascular and baroreflex function, subjects were asked to refrain from exercise, stimulants that might alter autonomic function (e.g., caffeine and cold medications including ephedrine, diphenhydramine), alcohol, prescription or non-prescription drugs, and herbal medications for 24 h prior to the orientation and experimental sessions. Subjects were also instructed to remain hydrated (ad libitum water consumption) and maintain their normal sleep pattern. Experiments were conducted at the same time of day (morning) to avoid potential effects of circadian rhythm on the study outcomes, in a temperature-controlled laboratory (22–24°C).

### Instrumentation

Subjects were placed in the supine position with their lower body inside a LBNP chamber (VUV Analytics, Austin, TX) and positioned on a bicycle seat to ensure they did not move during chamber decompression. Durable plastic and a neoprene band were wrapped around the subject’s waist to create an airtight seal with the LBNP chamber; the seal was in line with the subject’s iliac crest. All subjects were instrumented for the continuous measurement of HR via a standard lead II ECG (shielded leads, cable and amplifier, AD Instruments, Bella Vista, NSW, Australia), and beat-to-beat arterial pressure and SV via infrared finger photoplethysmography (Finometer, Finapres Medical Systems, Amsterdam, The Netherlands). Brachial arterial pressure recordings were made with a manual sphygmomanometer to verify the reconstructed finger arterial pressure readings from the Finometer. Respiration rate and end-tidal CO_2_ (etCO_2_) were measured on a breath-by-breath basis through a facemask (7940 Series, Hans Rudolph Inc., Shawnee, KS) via capnography (ML206 Gas Analyzer, AD Instruments, Bella Vista, NSW, Australia). Cerebral blood velocity was recorded from the MCAv and posterior cerebral artery (PCAv) via transcranial Doppler (TCD) ultrasound (2 MHz probes; ST3, Spencer Technologies, Seattle, WA) using standard techniques (e.g., Newell and Aaslid 1992). Oxygenated hemoglobin (HbO_2_), deoxygenated hemoglobin (dHb), total hemoglobin concentration (THC; HbO_2_ + dHb), and ScO_2_ [(HbO_2_/THC) * 100] were measured or calculated from the frontal cortex via near-infrared spectroscopy (NIRS, OxiplexTS, ISS Inc., Champaign-Urbana, IL). Selection of the side for the MCA probe was dependent on placement of the NIRS probe for assessment of cerebral oxygenation. The NIRS probe needed to be placed in a region of the forehead that was clear of hair, marks, blemishes, or discoloration. Once the side for the NIRS probe had been determined, the PCA probe was placed on the opposite side. For each repeated experiment, the same side of the head was used for the MCA and PCA probes. Within each subject, both MCAv and cerebral oxygenation measurements were made on same side of the head (*N* = 9 on the right side; *N* = 9 on the left side).

### Protocol

Each subject underwent two identical experimental sessions separated by at least 1 month, designated as Trial 1 and Trial 2. Four weeks intervened between experiments as all female subjects were tested in the early follicular phase of the menstrual cycle; males were also tested with at least 1 month intervening between trials to ensure consistency between all subjects, regardless of sex. These repeated trials were part of a larger study, so six subjects were exposed to an additional LBNP protocol with an acute intervention (inspiratory resistance breathing during LBNP) in between trials 1 and 2 described in the present investigation; trial 2 was always conducted at least 1 month following this protocol. The maximal LBNP protocol consisted of a 5-min baseline period followed by continuous application of negative pressure at a decompression rate of 3 mmHg/min (computer controlled at this set rate) until the presence of one or more of the following criteria: (1) instantaneous systolic arterial pressure (SAP) below 80 mmHg; (2) sudden relative bradycardia, and/or; (3) voluntary subject termination due to subjective presyncopal symptoms such as gray out, nausea, sweating, dizziness, blurred vision, or general discomfort. The chamber pressure was released immediately at the onset of hemodynamic decompensation or upon completing 1-min at −100 mmHg LBNP. Release of the chamber pressure occurred within seconds, and presyncopal symptoms generally resolved within 30–60 sec. Following LBNP termination, subjects remained in the chamber for a 10-min recovery period.

### Data analysis

All continuous waveform data (e.g., ECG, arterial blood pressure, SV, MCAv, ScO_2_, THC, etCO_2_) were collected at 1000 Hz (PowerLab and LabChart, AD Instruments, Bella Vista, NSW, Australia) and analyzed offline via specialized software (WinCPRS, Absolute Aliens, Turku, Finland). R-waves that were generated from the ECG signal were detected to determine the timing of each cardiac cycle. Beat-to-beat SAP and diastolic arterial pressures (DAP) were then detected from the continuous arterial pressure tracing. Systolic and diastolic cerebral blood velocities were also detected and marked from the continuous MCAv and PCAv tracings. MAP and mean MCAv and PCAv were automatically calculated as the area under the arterial pressure and cerebral blood velocity waveforms via the WinCPRS software. CO was calculated as the product of HR and SV; total peripheral resistance (TPR) was calculated as MAP divided by CO.

### Statistical analysis

All variables were analyzed from the final 4-min of each 5-min interval of LBNP. In addition, to compare physiological responses between Trial 1 and Trial 2 at presyncope, data was analyzed during the final 1-min prior to presyncope (PS-1). Pearson correlations were used to explore the relationship between time to presyncope (TTPS) between each trial, and all of the measured hemodynamic parameters during each LBNP exposure. Paired t-tests were also used to compare TTPS between trials, and the rate of change for all hemodynamic responses between trials (per mmHg LBNP, and per min). Two-way repeated measures ANOVAs were used to compare baseline and presyncopal hemodynamic responses across trials, followed by Tukey’s post hoc tests. Absolute and percentage change from baseline values are reported for the key variables of interest. All data are presented as mean ± SE (unless otherwise stated), and exact *P*-values are reported for all comparisons.

## Results

### LBNP tolerance

**Figure 1:**
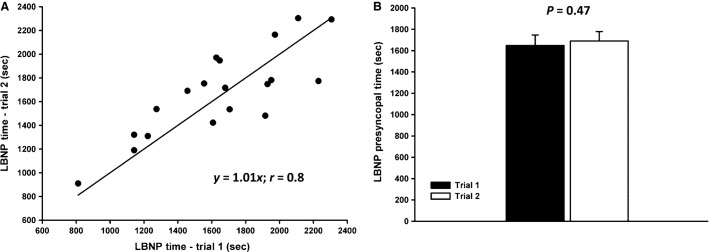
Panel (A): Correlation between time to presyncope for two trials of a ramp lower body negative pressure (LBNP) protocol (3 mmHg/min); solid line represents line of identity. Panel (B): Mean time to presyncope for Trial 1 (1649 ± 98 sec; black bar) and Trial 2 (1690 ± 88 sec; white bar) (*P* = 0.47).

Of the 27 subjects who completed both experimental trials, data was only analyzed and included for 18 subjects who (1) reached the maximal LBNP pressure (−100 mmHg), or; (2) had a minimum SAP < 80 mmHg, or; (3) exhibited subjective presyncopal symptoms combined with mean SAP < 100 mmHg for the entire 1-min prior to presyncope, and/or minimum SAP ≤ 90 mmHg within the 1-min prior to presyncope. Of these 18 subjects, LBNP was terminated upon reaching −100 mmHg LBNP for two subjects – one subject for both trials, and one subject for one of the two trials. There was no difference (*P* = 0.47) in TTPS between Trial 1 (1649 ± 98 sec) and Trial 2 (1690 ± 88 sec; slope = 1.01; *r* = 0.8) (Fig.1); the average coefficient of variation for TTPS was 8.5 ± 1.1%. Maximal LBNP pressures at presyncope were also linearly associated (slope = 1.01; *r* = 0.7) between Trial 1 (−68 ± 5 mmHg) and Trial 2 (−70 ± 4 mmHg; *P* = 0.47). Of the 6 subjects who participated in an additional LBNP protocol in between Trials 1 and 2 (as previously described), tolerance was not systematically higher in subjects exposed to three versus two LBNP protocols (*P* = 0.46). The minimum time between Trial 1 and 2 was 28 days, the maximum time was 119 days, and the average time between trials was 57 ± 7 days. In addition, each subject exhibited similar subjective presyncopal symptomology between trials (i.e., blurred vision, sweating, nausea, dizziness).

### Cardiovascular responses to LBNP

**Figure 2:**
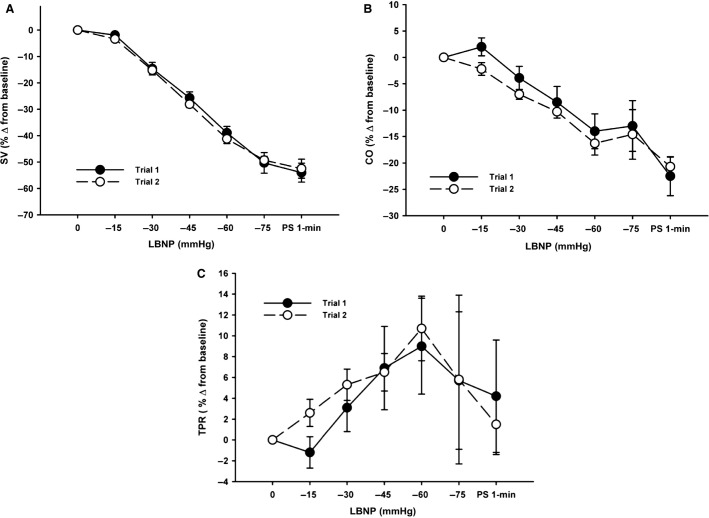
Percent change from baseline responses for stroke volume (SV, Panel A), cardiac output (CO, Panel B), and total peripheral resistance (TPR, Panel C) to a presyncopal-limited lower body negative pressure (LBNP) protocol for Trial 1 and Trial 2.

**Figure 3:**
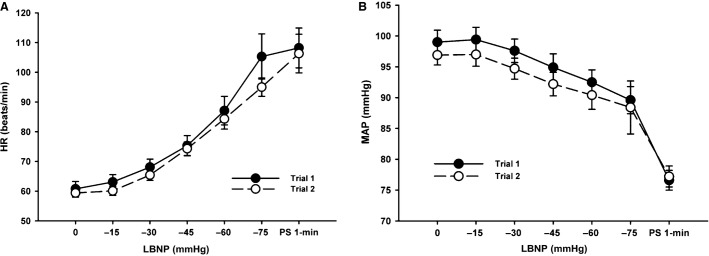
Heart rate (HR, Panel A) and mean arterial pressure (MAP, Panel B) responses to a presyncopal-limited lower body negative pressure (LBNP) protocol for Trial 1 and Trial 2.

**Figure 4:**
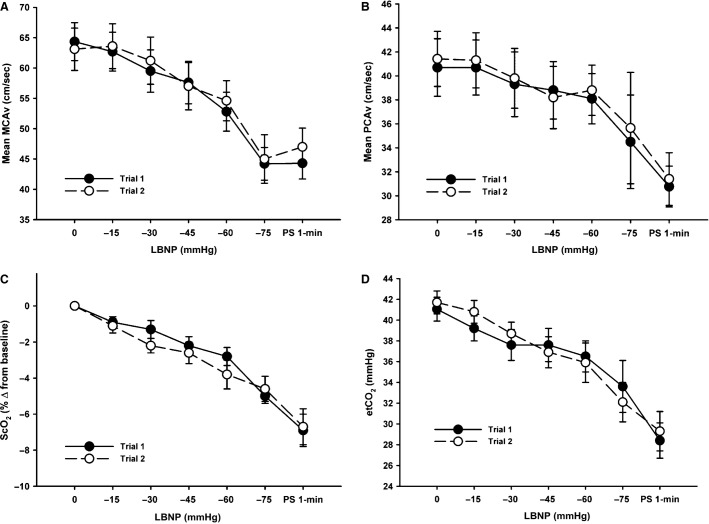
Mean middle cerebral artery velocity (MCAv, Panel A), mean posterior cerebral artery velocity (PCAv, Panel B), cerebral oxygen saturation (ScO_2_, % change from baseline, Panel C), and end-tidal carbon dioxide (etCO_2_, Panel D) responses to a presyncopal-limited lower body negative pressure (LBNP) protocol for Trial 1 and Trial 2.

Posterior cerebral artery (PCAv) data was only obtained on nine subjects through to presyncope for both trials. There were no statistically distinguishable differences in absolute HR, MAP, ScO_2_, MCAv, PCAv, and etCO_2_ between trials at baseline (*P* ≥ 0.17) or in the maximal responses at presyncope (*P* ≥ 0.19; Table1). As shown in Table1, Figures2 and 4, subjects also exhibited similar relative reductions in SV, CO, and ScO_2_, and increases in TPR at presyncope. As demonstrated in Figures2–4, all hemodynamic measurements of interest followed similar trajectories throughout LBNP for both trials, exhibiting high linear associations (Table2). There were also no statistically distinguishable differences in any of the measured parameters for rate of change per minute of the LBNP protocol (*P* ≥ 0.12) between trials, or the rate of change per mmHg of LBNP (*P* ≥ 0.12) (Table3).

**Table 1:** Comparison of physiological responses between baseline and presyncope (PS-1) within and between trials of presyncopal limited lower body negative pressure at a decompression rate of 3 mmHg/min

	Trial 1		Trial 2		Trial 1 versus Trial 2 *P*-values	
	Baseline	PS-1	Baseline	PS-1	Baseline	PS-1
HR (bpm)	60.8 ± 2.5	108.2 ± 6.7*	59.4 ± 1.4	106.3 ± 6.5*	0.70	0.60
MAP (mmHg)	99.0 ± 1.9	76.6 ± 1.6*	96.9 ± 1.6	77.2 ± 1.1*	0.17	0.66
SAP (mmHg)	132.3 ± 2.2	95.4 ± 1.3	130.1 ± 1.9	96.3 ± 1.6	0.22	0.59
DAP (mmHg)	76.5 ± 1.7	65.6 ± 1.8	74.8 ± 1.4	66.0 ± 1.8	0.28	0.82
SV (% ∆)	–	−54.0 ± 3.6*	–	−52.5 ± 3.6*	–	0.40
CO (% ∆)	–	−22.5 ± 3.7*	–	−20.7 ± 1.8*	–	0.34
TPR (% ∆)	–	4.2 ± 5.4	–	1.5 ± 2.9	–	0.40
etCO_2_ (mmHg)	41.0 ± 1.1	28.4 ± 1.7*	41.7 ± 1.1	29.3 ± 1.9*	0.69	0.56
MCAv (cm/s)	64.4 ± 3.1	44.3 ± 2.6*	63.1 ± 3.5	47.0 ± 3.1*	0.50	0.19
PCAv (cm/s)	40.7 ± 2.4	30.8 ± 1.7*	41.1 ± 2.3	31.4 ± 2.2*	0.70	0.75
ScO_2_ (%)	67.3 ± 1.7	62.7 ± 1.6*	67.0 ± 1.6	62.5 ± 1.6*	0.69	0.79

Data are presented as means ± SE. HR, heart rate; MAP, mean arterial pressure; SV, stroke volume; CO, cardiac output; TPR, total peripheral resistance; etCO_2_, end tidal carbon dioxide; MCAv, middle cerebral artery velocity; PCAv, posterior cerebral artery velocity; ScO_2_, cerebral oxygen saturation. Baseline and presyncopal (PS-1) responses were compared between Trial 1 and Trial 2.

* *P* < 0.001 between baseline and PS-1 within a trial. *N* = 18; *N* = 9 for PCA.

**Table 2:** Correlation data for physiological responses between Trial 1 and Trial 2 with continuous application of lower body negative pressure at a decompression rate of 3 mmHg/min

Parameter	Slope	Correlation coefficient (*r*)	*P*-value
HR (bpm)	1.07	0.99	<0.001
MAP (mmHg)	1.16	0.99	<0.001
SAP (mmHg)	1.09	0.99	<0.001
DAP (mmHg)	1.17	0.98	<0.001
SV (% ∆)	1.03	0.99	<0.001
CO (% ∆)	1.12	0.98	<0.001
TPR (% ∆)	0.85	0.83	0.02
etCO_2_ (mmHg)	0.89	0.97	<0.001
MCAv (cm/s)	1.09	0.99	<0.001
PCAv (cm/s)	1.02	0.99	<0.001
ScO_2_ (% ∆)	1.06	0.98	<0.001

Data are presented as means ± SE. HR, heart rate; MAP, mean arterial pressure; SV, stroke volume; CO, cardiac output; TPR, total peripheral resistance; etCO_2_, end tidal carbon dioxide; MCAv, middle cerebral artery velocity; PCAv, posterior cerebral artery velocity; ScO_2_, cerebral oxygen saturation. *N* = 18; *N* = 9 for PCA.

**Table 3:** Rate of change (per minute and per mmHg LBNP) from baseline to presyncope for all physiological variables for the two trials

Parameter	Rate of change (per min)			Rate of change (per mmHg)		
	Trial 1	Trial 2	*P*-value	Trial 1	Trial 2	*P*-value
HR (bpm)	1.61 ± 0.15	1.56 ± 0.18	0.71	0.65 ± 0.06	0.63 ± 0.07	0.63
MAP (mmHg)	−0.85 ± 0.09	−0.73 ± 0.07	0.15	−0.36 ± 0.04	−0.30 ± 0.03	0.13
SAP (mmHg)	−1.38 ± 0.09	−1.22 ± 0.09	0.15	−0.58 ± 0.04	−0.50 ± 0.04	0.12
DAP (mmHg)	−0.43 ± 0.07	−0.34 ± 0.06	0.26	−0.18 ± 0.03	−0.14 ± 0.03	0.25
SV (mL)	−2.08 ± 0.16	−1.90 ± 0.11	0.19	−0.86 ± 0.08	−0.77 ± 0.05	0.15
CO (L/min)	−0.05 ± 0.01	−0.05 ± 0.004	0.23	−0.02 ± 0.004	−0.02 ± 0.002	0.20
TPR (mmHg/L/min)	0.03 ± 0.04	0.001 ± 0.02	0.43	0.01 ± 0.02	0.00002 ± 0.007	0.42
etCO_2_ (mmHg)	−0.47 ± 0.06	−0.43 ± 0.05	0.61	−0.19 ± 0.03	−0.17 ± 0.02	0.53
MCAv (cm/s)	−0.72 ± 0.08	−0.56 ± 0.08	0.12	−0.30 ± 0.04	−0.23 ± 0.04	0.12
PCAv (cm/s)	−0.35 ± 0.08	−0.35 ± 0.01	0.99	−0.14 ± 0.04	−0.14 ± 0.01	0.92
ScO_2_ (%)	−0.16 ± 0.02	−0.15 ± 0.02	0.64	−0.07 ± 0.01	0.06 ± 0.01	0.53

Data are presented as means ± SE. HR, heart rate; MAP, mean arterial pressure; SV, stroke volume; CO, cardiac output; TPR, total peripheral resistance; etCO_2_, end tidal carbon dioxide; MCAv, middle cerebral artery velocity; PCAv, posterior cerebral artery velocity; ScO_2_, cerebral oxygen saturation. *N* = 18; *N* = 9 for PCA.

## Discussion

In this study, we examined the reproducibility of continuous application of LBNP to presyncope at a rate of 3 mmHg/min in a cohort of young, healthy human subjects. The key findings demonstrate that (1) time to presyncope is reproducible (moderate to high) with application of ramp-LBNP at a decompression rate of 3 mmHg/min; (2) maximal stroke volume reduction (50–55%) was similar between trials; and (3) all reflex physiological responses were highly reproducible. As there were no statistically distinguishable differences between either baseline or presyncopal values for any of the hemodynamic parameters explored in this study, subjects appeared to be in a similar physiological state at rest, and the presyncopal state was represented by reproducible physiological responses.

While a number of studies have assessed the reproducibility of the traditional stepwise LBNP pressure profile (Lightfoot et al. 1991; Convertino 2001; Howden et al. 2001), none, to the best of our knowledge, have examined the reproducibility of a continuous ramp-LBNP pressure profile. Since the introduction of LBNP as a research tool in the 1960s (Stevens and Lamb 1965; Brown et al. 1966), many laboratories have adopted this technique for the investigation of physiological responses to variations in central blood volume, using both cross-sectional and interventional experimental designs. The majority of investigators utilize step-LBNP protocols, but vary the profiles in terms of the magnitude and length (time) of each pressure step, and the termination point, which is generally limited by either the subject (i.e., presyncope or discomfort), or the physical capability of the LBNP chamber (i.e., maximum pressure). In those investigations that have explored the reproducibility of step-LBNP, different pressure profiles have been used, and the time separation between repeated LBNP exposures has varied, from days up to a year. Howden et al. tested the reproducibility of a stepwise pressure profile in subjects who underwent LBNP to tolerance on 3 occasions, each separated by 72–120 h (3–5 days) (Howden et al. 2001). These investigators demonstrated that there were no differences in HR, or arterial pressure (SAP, DAP) responses during maximal LBNP to presyncope (*P* ≥ 0.31) when retesting subjects three times (Howden et al. 2001). However, there was a difference in tolerance assessed via calculation of the LBNP tolerance index (LTI), and the cumulative stress index (CSI); tolerance between trial 1 and 2 was similar, but tolerance for trial 3 was higher than both trial 1 and 2 (Howden et al. 2001). Conversely, other investigators have shown that tolerance (TTPS in min) does not change between four LBNP exposures separated by at least 72 h (Lightfoot et al. 1991), or between two trials with 1-year intervening (*r* = 0.94) (Convertino 2001). Lightfoot et al. did demonstrate, however, that the reproducibility of LBNP tolerance improves with repeated exposures, and exposures closer in time (*r* = 0.71 for test 1 vs. test 2, *r* = 0.97 for test 2 vs. test 3, *r* = 0.93 for test 3 vs. test 4) (Lightfoot et al. 1991). The findings from our study shows similar reproducibility using the ramp pressure profile (*r* = 0.77), where tolerance to repeated presyncopal-limited LBNP exposures (indexed by TTPS) was assessed in the same subjects separated by at least 1 month (Range: 30–119 days). In agreement with previous studies utilizing step-LBNP protocols, we also demonstrated the reproducibility of HR, SAP, DAP, and MAP responses. This study is also novel in examining the reproducibility of MCAv, PCAv, and ScO_2_ responses, which has not been assessed during any LBNP protocol (step or ramp). Continued use of ramp-LBNP for investigation of cerebral blood velocity and oxygenation responses to experimental central hypovolemia is warranted based on these findings.

Adaptation to LBNP and variability in tolerance to central hypovolemia are important factors to consider when subjecting individuals to repeated exposures of presyncopal-limited LBNP. Multiple studies, including the present investigation, have shown that tolerance to LBNP is variable, such that subjects become presyncopal at different magnitudes of central hypovolemia (Sather et al. 1986; Levine et al. 1994; Greenleaf et al. 2000; Rickards et al. 2011). Although tolerance may vary from subject to subject, tolerance within an individual subject over multiple exposures of LBNP appears to be similar. The time between multiple LBNP exposures is also an important consideration when designing studies using this technique. Lightfoot et al. (Lightfoot et al. 1989) explored potential physiological adaption to LBNP by exposing subjects to stepwise presyncopal-limited LBNP every day for 9 days, with a 2-day break between days 5 and 6. LBNP tolerance progressively increased over the course of nine daily LBNP exposures, by a maximum of 49% from day one, equating to an increase in tolerance duration of 6.5 min. On day 6, even with a 2-day break, LBNP tolerance remained at day 5 levels (Lightfoot et al. 1989). These investigators speculated that repeated exposures to a central hypovolemic stress may cause acute resetting of the baroreflex, allowing for more effective cardiovascular compensation during subsequent hypotensive stress resulting in improved tolerance (Lightfoot et al. 1989). As such, Lightfoot et al. concluded that more than 2 days should intervene between repeated LBNP exposures to avoid the risk of cardiovascular adaptation; we are confident that the minimum 4-week interval between trials used in this study was sufficient to avoid any physiological adaptation.

### Methodological considerations

This study assessed the reproducibility of a novel ramp-LBNP protocol in 18 healthy human subjects. This is a relatively small sample size, and only one-third of these subjects were female. Additional testing is warranted in a larger sample of female subjects to make meaningful statistical comparisons between males and females, and across different phases of the menstrual cycle within female subjects. Furthermore, we tested the reproducibility to just two trials of this ramp-LBNP profile. Additional studies could compare responses to more than 2 trials to determine if tolerance to this stress is progressively more reproducible as the number of trials increases, as previously demonstrated with step-LBNP protocols (see discussion above) (Lightfoot et al. 1991).

## Conclusions

The findings from this study indicate that ramp LBNP applied at an onset rate of 3 mmHg/min is reproducible in terms of tolerance time and hemodynamic responses, so could be used as a reliable method for assessment of cardiovascular and cerebrovascular responses to central hypovolemia. The continuous nature of the decompression profile may more accurately simulate actual blood loss, although direct comparison of responses to actual hemorrhage versus continuous LBNP is required to adequately address this hypothesis.
